# Preparation, Characterization and in Vivo Antimycobacterial Studies of Panchovillin-Chitosan Nanocomposites

**DOI:** 10.3390/ijms17101559

**Published:** 2016-09-27

**Authors:** Edward Rwegasila, Egid B. Mubofu, Stephen S. Nyandoro, Paul Erasto, Joan J. E. Munissi

**Affiliations:** 1 Chemistry Department, College of Natural and Applied Sciences, University of Dar es Salaam, P.O. Box 35061, 14115 Dar es Salaam, Tanzania; edrwega@yahoo.com (E.R.); ebmubofu@udsm.ac.tz (E.B.M.); nyandoro@udsm.ac.tz (S.S.N.); 2 National Institute for Medical Research (NIMR), P.O. Box 9653, 14115 Dar es Salaam, Tanzania; paulkazyoba@yahoo.co.uk

**Keywords:** panchovillin, chitosan, nanocomposites, antimycobacterial, *Galleria mellonella*

## Abstract

Chitosan (CS, molecular weight 20.2 kDa, degree of deacylation (DD) 73.31%) was successfully obtained by deacetylation of chitin extracted from shrimp (*Litopenaeus vannamei*) shell wastes. The encapsulation of the bioactive natural product, panchovillin (PANV), isolated from *Erythrina schliebenii*, on a chitosan-tripolyphosphate (CS/TPP) nano-framework was achieved by ionotropic gelation. Characterization of pure CS, CS/TPP and PANV-CS/TPP nanocomposites was performed by FTIR, SEM and XRD. The molecular weight of chitosan and the thermal stability of the materials were determined by MALDI-TOF-MS and simultaneous thermal analyzer (STA)/DTG, respectively. The respective encapsulation efficiency and loading capacity of the PANV were found to be 70% and 0.36%. The in vitro release studies showed an initial burst of 42% of PANV in the first six hours. This was followed by a slow and sustained release up to 72 h. The in vivo antimycobacterial activities of both PANV and PANV-CS/TPP nanocomposite against *Mycobacterium indicus pranii* (MIP) using *Galleria mellonella* larvae as an in vivo infection model are reported in this paper.

## 1. Introduction

Nanotechnology is a multidisciplinary field that employs proficiencies and tools from diverse disciplines [1]. The technology has offered tremendous advancement in the field of therapeutics by means of designing various drug delivery systems, thereby increasing the possibilities of controlling infections at the molecular level [2]. The nanoparticle-based systems exhibit significant potential for treatment and prevention of tuberculosis due to their ability to intersect biological barriers and targeting the cellular reservoirs of *Mycobacterium tuberculosis* (MTB) [2]. Appreciable interest in the search for potential biomedical applications of biomaterials, such as exosomes, liposomes and chitosan, as carriers of therapeutic agents has been reported [1,3]. Over the years, interest in chitosan has dramatically grown due to its novel properties, such as biocompatibility, biodegradability, affordability, stability, low toxicity, as well as its simple and moderate preparation procedures. Moreover, chitosan offers various administration routes, such as oral, nasal and ocular mucosa, making it one of the most significantly attractive materials for drug delivery [4,5]. Chitosan has been investigated as a carrier of bioactive molecules to various target cells, such as cancer cells [6]. However, scanty information is available on the application of chitosan in the delivery of potential anti-TB drugs. The treatment of tuberculosis currently involves the use of first line anti-tubercular drugs, such as rifampicin, isoniazid, ethambutol and pyrazinamide [7]. Longer treatment periods (approximately six to twelve months) have always posed a threat of attrition to treatment, leading to the development of drug-resistant TB strains. Thus, the application of nanotechnology to improve drug delivery and bioavailability is likely to reduce the treatment time and improve compliance and outcome [1,8]. Natural products possessing inhibitory activity against MTB have been reported from higher and lower forms of plants, microorganisms and marine organisms [9]. In most cases, natural products are rendered inactive in vivo due to low solubility, poor stability, low bioavailability and lack of target specificity, leading to inaccessibility at their most effective levels at the target site. Thus, formulating delivery systems that optimize the effectiveness of the compounds while minimizing their side effects is a crucial means of addressing the challenges associated with the treatment of MTB.

**Figure 1:**
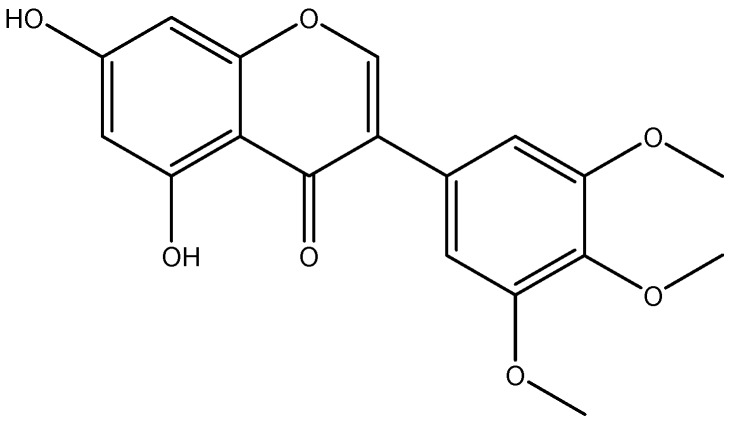
Chemical structure of panchovillin (PANV).

Polyphenols are economically important compounds that have found various applications, such as natural additives, food complements or have been incorporated into cosmetic or pharmaceutical formulations. However, the application of polyphenols is limited [10]; due to poor bioavailability arising from insufficient gastric residence time, low permeability and/or low solubility. These factors limit the activity and the potential health benefits of most polyphenols. Their tendency to oxidize easily in their free form leads to a considerable loss in activity. Therefore, the optimal exploitation and application of these compounds require that the drawbacks are circumvented in order to ensure that they reach a physiological target at desirable concentrations. This study therefore aimed at investigating the potential of improving the efficacy of panchovillin by encapsulation onto chitosan. Panchovillin (PANV; Figure 1) is a naturally-occurring polyphenol that has been reported from a variety of plant species, including *Erythrina schliebenii*, a rare and critically endangered plant species endemic to Tanzania [11]. The compound has been found to possess in vitro activities against *Mycobacterium tuberculosis* (H37Rv) and cytotoxicity against the aggressive human breast cancer cells (MDA-MB-231) [12]. Panchovillin was therefore selected as a representative polyphenol, while *Mycobacterium indicus pranii* (MIP) was chosen as a test organism using *Galleria mellonella* larvae as in vivo infection model to study the antimycobacterial properties of PANV and PANV-chitosan (CS)/tripolyphosphate (TPP) nanocomposites.

## 2. Results and Discussion

### 2.1. IR Analysis

**Figure 2:**
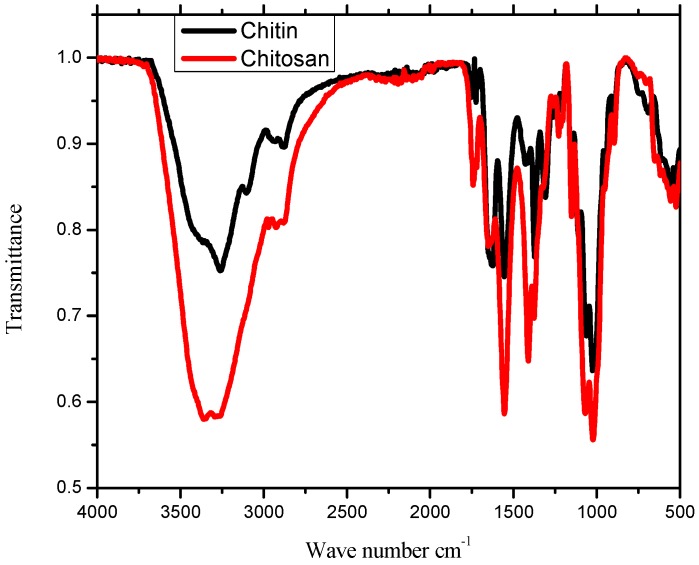
IR spectra of chitin and chitosan.

The IR spectra of chitin and chitosan are shown in Figure 2. The IR spectrum of chitin (Figure 2) was characterized by three important amide bands at 1624, 1555 and 1308 cm^−1^, which correspond to the stretching vibration of C=O, the bending vibration of the amide II band and C–N stretching vibration, respectively [13]. Other absorption bands observed at 3261, 2878 and 1153 cm^−1^ were attributed to O–H, C–H and C–O stretching vibrations, respectively, thereby confirming that the purified material was indeed chitin [14]. The infra-red spectrum of chitosan (Figure 2) exhibited a characteristic band at 3361 cm^−1^ attributed to the stretching vibration of the inter- and intra-molecular hydrogen bonds from the –NH_2_ and –OH groups, being similar to the previously-reported results [15]. The weak absorption band at 2925 cm^−1^ was assigned to *sp*^3^ C–H stretching vibrations. The intense peak around 1555 cm^−1^ corresponds to the bending vibration of NH_2_, a significant feature for chitosan. The higher intensity of this peak in chitosan compared to that observed in chitin and subsequent shortening of the peak intensity due to C=O stretch at 1651 cm^−1^ (Figure 2) indicates that deacetylation was successful [16,17]. The peak for the asymmetric stretch of C–O–C was observed at 1151 cm^−1^ [15,18,19]. The peaks at 1651 and 1372 cm^−1^ were attributed to C=O and C–N stretching vibration as amide I and amide III of chitosan, respectively [15].

**Figure 3:**
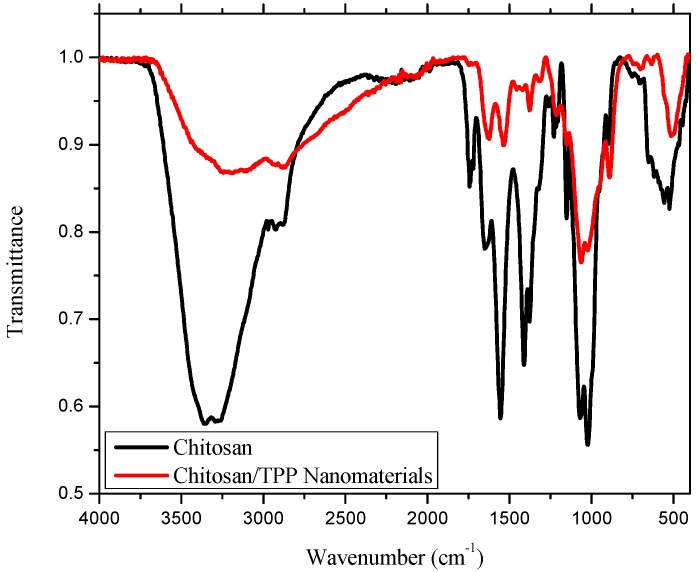
IR spectra of chitosan (CS) and CS/tripolyphosphate (TPP) nanomaterials.

Formation of CS/TPP nanomaterial was confirmed by comparing the IR spectra of pure CS and CS/TPP nanomaterial (Figure 3). The IR spectra revealed the peak at 3361 cm^−1^ in pure CS that had shifted to 3204 cm^−1^ in CS/TPP nanomaterial (Figure 3) and broadened with decreased relative intensity at a ratio of 3:1 (A_3361_/A_3204_), manifesting an enhancement of hydrogen bonding [19]. The peak for the N-H bending vibration of amide IІ at 1555 cm^−1^ in CS had shifted to 1535 cm^−1^ in CS/TPP nanomaterial with relative reduced intensity at approximately a 5:1 ratio (A_1555_/A_1535_), while the amide І C=O stretch observed at 1651 cm^−1^ in CS shifted to 1625 cm^−1^ in CS/TPP (Figure 3). A new peak observed at 1214 cm^−1^ was attributed to a P=O stretching vibration, confirming a cross-linkage between CS and TPP during fabrication of the nanomaterials [19,20].

**Figure 4:**
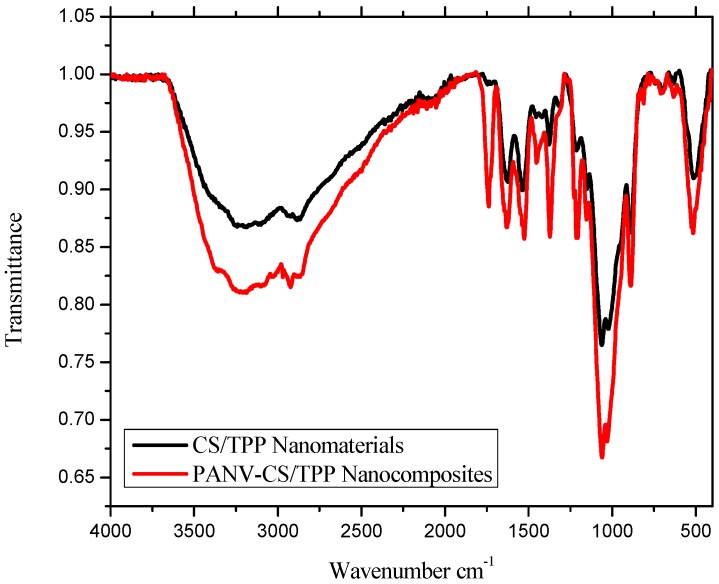
FTIR spectra of free CS/TPP nanomaterials and PANV-CS/TPP nanocomposites.

**Figure 5:**
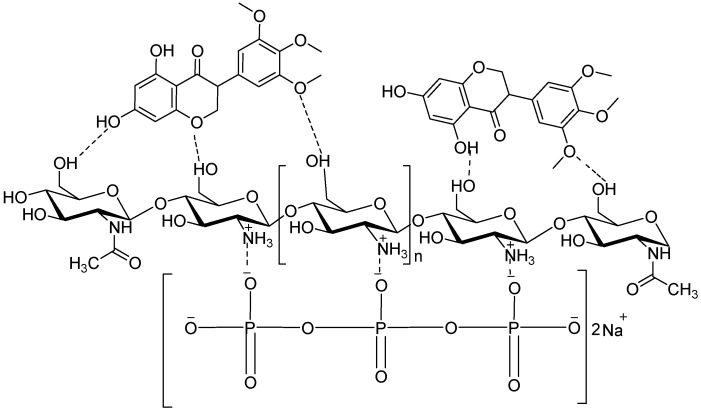
Hypothetical interactions between of CS/TPP and PANV.

The IR spectrum of the PANV-CS/TPP nanocomposites showed a shift of absorption peaks when compared to the free chitosan nanomaterials (Figure 4). The absorption peak observed at 3208 cm^−1^ corresponds to OH and N–H, while other peaks at 1621, 1523, 1215 and 1155 cm^−1^ were assigned to the amide I (C=O) band, the amide II band, the P=O stretch and the asymmetric stretch of C–O, respectively (Figure 4). The new peak observed at 1733 cm^−1^ is due to the ketonic C=O stretch of PANV, confirming the successful encapsulation of the compound on the CS/TPP nano-framework. The encapsulation of PANV within the CS/TPP framework is probably enhanced by the hydrogen bonding between the methoxy and hydroxyl groups present in PANV and the hydroxyl groups on the polymer (Figure 5).

### 2.2. Thermal Analysis

**Figure 6:**
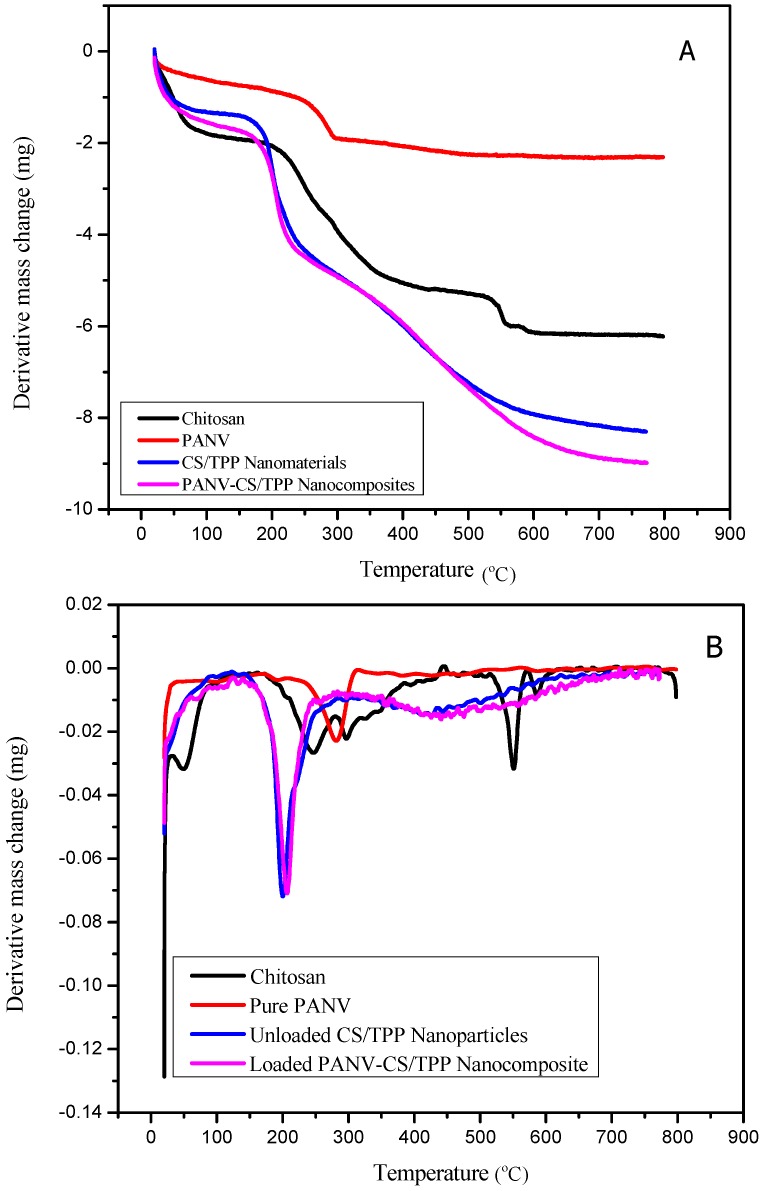
(**A**) Simultaneous thermal analysis (STA) curves of chitosan, PANV, free CS/TPP nanomaterials and PANV-CS/TPP nanocomposites; (**B**) Differential thermal gravimetric (DTG) curves of chitosan, PANV, free CS/TPP nanomaterials and PANV-CS/TPP nanocomposites.

The thermal decomposition and stability of CS, CS/TPP, PANV and PANV-CS/TPP are depicted in the simultaneous thermal analyzer (STA)/DTG curves shown in Figure 6A,B. The STA/DTG curve of chitosan (Figure 6A) shows the first thermal event that occurred in the temperature range of 50 to 85 °C being associated with 27.6% weight loss assigned to dehydration [15,21,22,23,24,25,26]. The second event of thermal decomposition observed from 210 to 390 °C was associated with 50.1% weight loss due to degradation of the chitosan polymer. Moreover, a decomposition from 391 to 600 °C at 2.5% weight loss is associated with the decomposition of constituting acetic and butyric acids and a series of lower fatty acids [24,25]. Finally, from 600 to 800 °C, a constant curve was observed accomplishing a total loss of 80.4% of the initial amount. The PANV alone has lower stability than CS and CS/TPP nanomaterials (Figure 6A) that showed comparatively higher stabilities. The STA/DTG curve of CS/TPP (Figure 6A,B) shows the first thermal effect from 50 to 100 °C being due to the removal of water molecules. The free CS/TPP was thermally stable at temperatures below 200 °C, this being the temperature at which the decomposition of chitosan was observed to occur (Figure 6B). Further decomposition associated with the vaporization and elimination of volatile products was observed around 300 °C until it reached the final stability, notably from 600 to 800 °C [27,28]. On the other hand, the cross-linking reaction through TPP modified the crystalline nature of chitosan since the decomposition of CS/TPP was reduced to two stages (Figure 6A) compared to the observed three stages for the pure chitosan.

The STA/DTG curve of the PANV-CS/TPP (Figure 6A,B) shows a slight difference in thermal stability compared to the free CS/TPP. After dehydration at 100 °C, PAN-CS/TPP kept decomposing to about 22% at 196 °C. The final decomposition event was observed from 262 to 600 °C, which is accompanied by the dissociation of pure PANV (Figure 6A).

### 2.3. SEM and X-ray Diffraction

**Figure 7:**
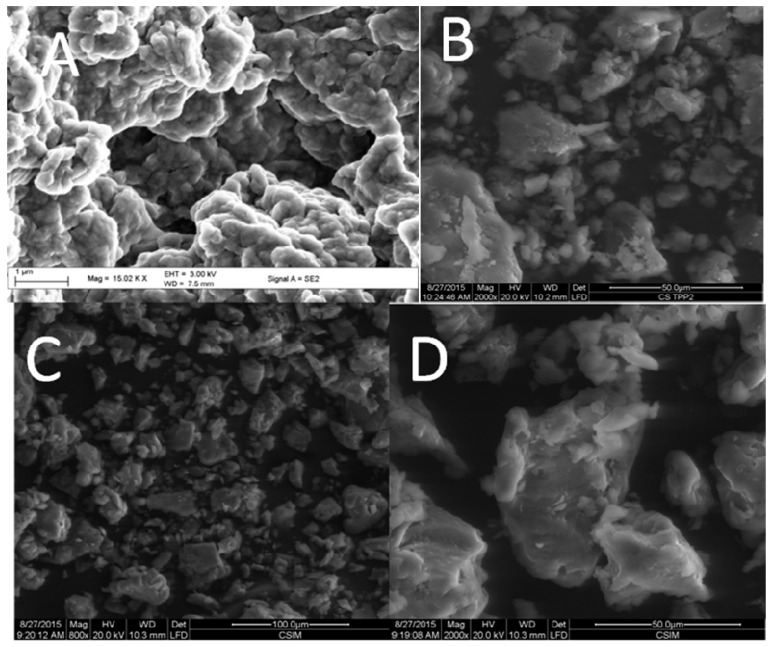
SEM images of (**A**) chitosan (scale bar = 1 μm); (**B**) CS/TPP (scale bar = 50 μm); (**C**) PANV-CS/TPP (scale bar = 100 μm); and (**D**) PANV-CS/TPP (scale bar = 50 μm).

The scanning electron micrographs of pure chitosan showed an uneven and rough surface with a spongy appearance (Figure 7A). The rough surface of the chitosan is attributable to the low degree of deacetylation [29]. The SEM image (Figure 7B) of CS/TPP indicated non-uniform agglomerated particles with no clearly defined morphology. Agglomeration was also vivid when the bioactive compound was encapsulated, leading to the formation of a more compact non-uniform nanoformulation, as shown in the SEM images (Figure 7C,D). The particle sizes of CS/TPP materials ranged from 1.128 to 200 μm while those of PANV-CS/TPP nanocomposites had diameters ranging from 0.359 to 12 μm. The strong interaction caused by inter- and intra-molecular hydrogen bonding between the polyphenol and chitosan during the fabrication of CS/TPP-loaded nanocomposites might have contributed to the observed reduced size of the agglomerated particles (Figure 7C).

**Figure 8:**
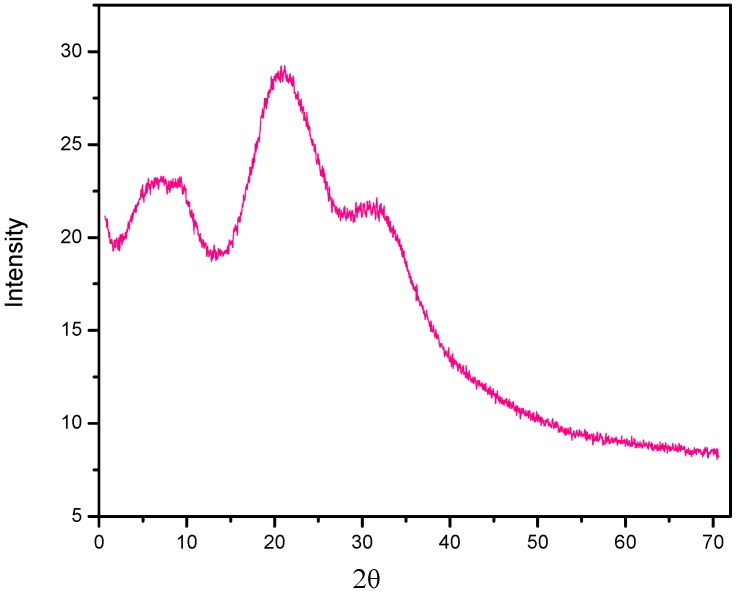
X-ray diffractogram of CS/TPP.

The powder X-ray diffraction patterns of CS/TPP (Figure 8) depicted the crystalline nature of the particles with intense reflections at 2θ = 8.15° and 20.93°, together with minor reflection 2θ at 30.33°. The XRD of the PANV-CS/TPP nanocomposite was not recorded due to sample limitations.

### 2.4. Effect of PANV and Chitosan Concentrations on Encapsulation Efficiency

**Figure 9:**
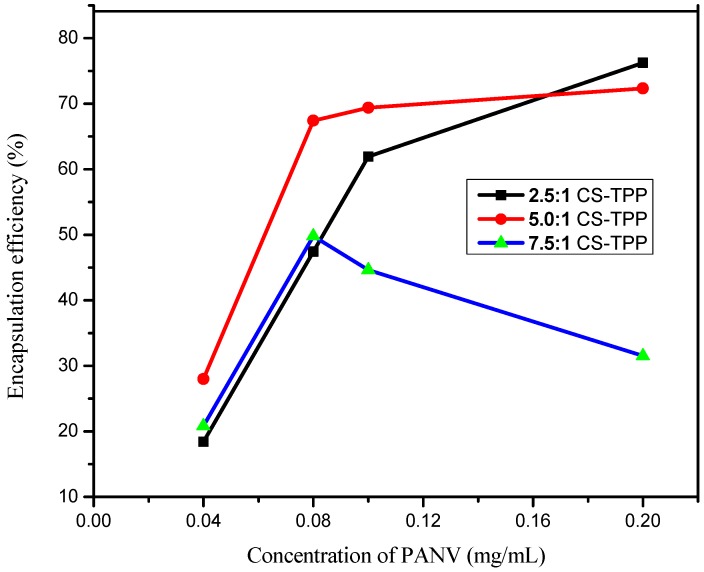
The effect of the PANV concentration on encapsulation efficiency.

The effect of the test compound concentrations (0.04, 0.08, 0.1 and 0.2 mg/mL) and chitosan concentrations (1, 2 and 3 mg/mL) was evaluated to determine the encapsulation efficiency (EE). The results summarized in Figure 9 show that the increase of panchovillin concentrations from 0.04 to 0.2 mg/mL brought an increased EE under different chitosan concentrations. This effect is interpreted as the outcome of the improved intercalation of the polyphenol into the chitosan network ensuing an activation of hydroxyl sites and establishing stronger hydrogen bonding in the system [30]. A smooth increase in EE was observed when the 1 and 2 mg/mL chitosan solutions were used as compared to the chitosan solution of a 3 mg/mL concentration, which indicated a tremendous decrease in EE (Figure 9). This enormous decrease in EE with the increase in chitosan solution concentration might be due to the high viscosity of the gelation medium with a high concentration of chitosan, resulting in a decrease in the liquid phase resistance against dispersion and, hence, forming large nanoparticles and a further decrease in encapsulation [20]. The encapsulation efficiency decreased from 76.24% to 31.52% as the CS/TPP mass ratio increased from 2.5:1 to 7.5:1. Therefore, small CS/TPP ratios of 2.5:1 and 5.0:1 associated with low gelation medium and a low concentration of chitosan solution are suitable for higher encapsulation efficiency than a higher CS/TPP mass ratio of 7.5:1. A similar trend has previously been reported elsewhere [30]. Overall, the CS/TPP ratio of 2.5:1 is the best because it achieves higher EE and, hence, higher concentrations of the polyphenol, panchovillin. However, for further studies that included the in vitro release kinetics, the in vivo antimycobacterial assay and physical characterizations, the 5.0:1 CS/TPP ratio was preferred, for which the EE and loading capacity (LC) were found to be 70.12% and 0.36%, respectively.

### 2.5. In Vitro Release Studies of PANV-CS/TPP

**Figure 10:**
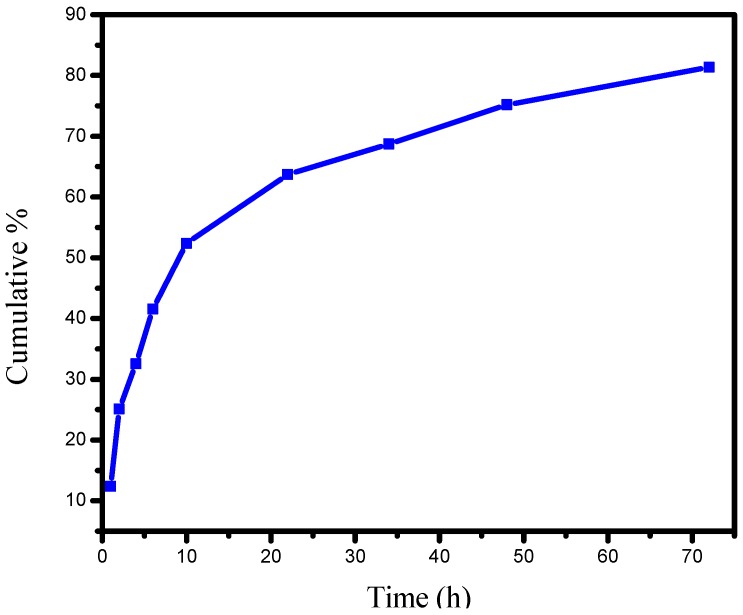
The release profiles of PANV-CS/TPP prepared using a 5.0:1 CS/TPP mass ratio.

The in vitro release profile of the PANV-CS/TPP as prepared using the 5.0:1 CS/TPP solution is indicated in Figure 10. The in vitro release profile shows the initial burst of 42% of the PANV after six hours. The initial burst release is conceived to be due to the poorly-entrapped and adsorbed bioactive compound when it interacts with chitosan during the fabrication of the nanoformulation. The study further concluded that 81% of PANV was released within 72 h. The burst release observed in the present study at 6 h (Figure 10) could be desirable for the initial release of a high amount of the bioactive compounds with the ensuing quick and effective availability within 6 h.

### 2.6. In Vivo Antimycobacterial Assay

**Figure 11:**
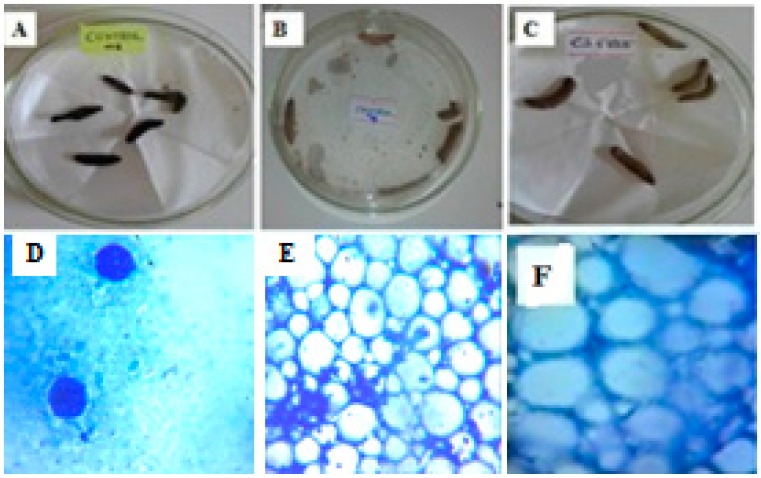
(**A**,**D**) Infected larvae (Positive control) and their appearance under the microscope at the magnification of 100×; (**B**,**E**) Non-infected Larvae (Negative control) and their appearance under the microscope at the magnification of 100×; (**C**,**F**) Larvae injected with Free CS/TPP and their appearance under the microscope at the magnification of 100×.

**Figure 12:**
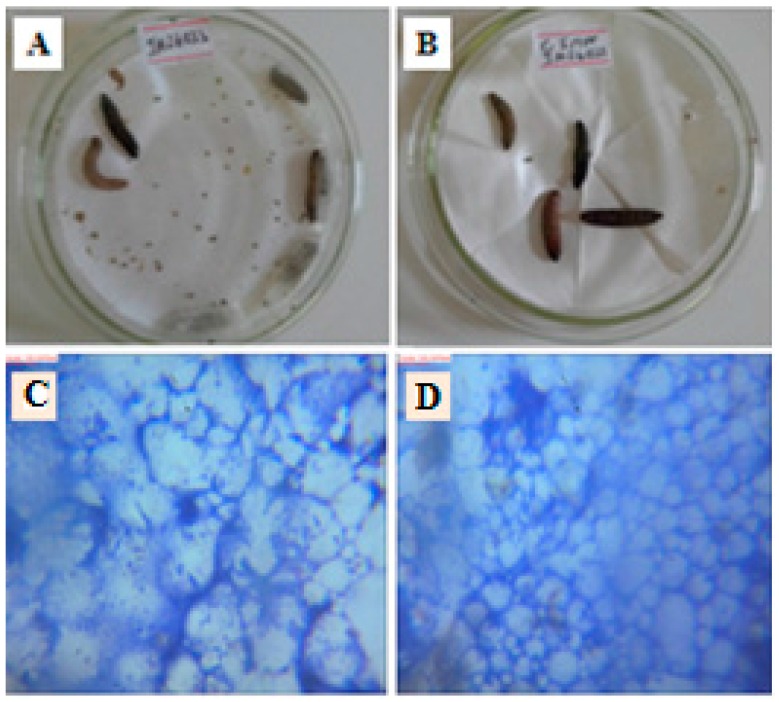
(**A**) Larva treated with the PANV and their appearance under the microscope at the magnification of 100× (**C**); (**B**) Larva treated with the PANV-CS/TPP nanocomposites and their appearance under the microscope at the magnification of 100× (**D**).

The in vivo antimycobacterial results are presented in Table 1 and in Figure 11 and Figure 12. The behaviors and effects of larvae treated with PANV and PANV-CS/TPP were observed by comparing with the controls after 24 and 48 h (Table 1 and Figure 12). The positive control larvae were injected with MIP only, and the body immune system response against the introduced bacteria was observed. The inoculated larvae turned into a black color after ten minutes, indicating the larval immune reaction due to cleavage of prophenoloxidase to activate phenoloxidase that limit the growth of MIP, as similarly reported elsewhere [31]. All of the positive control larvae died and appeared black colored after 24 h of incubation at 37 °C (Table 1 and Figure 11A). The death of positive control larvae resulted from body immune response failure to fight against the bacteria. This resulted in the extreme growth of bacteria colonies, which were highly concentrated in the cells of the infected larva (Figure 11D). The negative control larvae (Figure 11B) were not injected with bacteria and survived to the end of the study (48 h), proving the absence of infection. This was established beyond a doubt, as their cells were clean and well differentiated as observed under the microscope (at the magnification of 100×), indicating the absence of pathogen colonies (Figure 11E). The results obtained when the larvae were injected with free chitosan nanomaterials (Table 1 and Figure 11C,F) showed the larvae to be active throughout the study, manifesting the non-toxicity of chitosan.

The in vivo antimycobacterial results of PANV and PANV-CS/TPP were slightly different (Table 1 and Figure 12). This was further confirmed statistically, whereby after 24 h, the difference between the larvae treated with PANV and PANV-CS/TPP was not significant at *p* < 0.05 (χ^2^-statistcs of 1.2757, *p*-value = 0.258). However, a clear difference was observed after 48 hours, whereby PANV prolonged the life of larvae by 70% compared to the PANV-CS/TPP. The difference between the two treatment was statistically significant at *p* < 0.05 with the χ^2^-statistcs of 19.455 and *p*-value = 0.00001.

Furthermore, the clearance of bacteria colony density as observed in Figure 12C,D compared to the positive control in Figure 11D and the negative control in Figure 11E indicates that the PANV retained its antimycobacterial efficacies, even when applied to an in vivo system. This is contrary to most natural products that tend to lose their efficacies in in vivo models [32]. It is important to note that the amount of free PANV tested was far higher than the amount of PANV loaded on CS/TPP. The concentration of encapsulated PANV injected into the infected larva was just 0.289 nM/mg body weight compared to free PANV, which was 80 nM/mg body weight. Therefore, although PANV appeared to be more efficacious than PANV-CS/TPP, still, the latter was relatively more efficacious than the former. This assay has thus proven that the efficacy of PANV was indeed enhanced when encapsulated in the CS nanomaterials.

## 3. Materials and Methods

### 3.1. Test Organisms

The non-pathogenic *Mycobacterium indicus pranii* (MIP) strain DSM 45239 supplied by DSMZ (The Germany Resource Centre for Biological Materials, Braunschweig, Germany) was used to determine of anti-mycobacterial properties of the free panchovillin and panchovillin-loaded chitosan nanocomposites.

### 3.2. Reagents and Other Materials

All chemicals and reagents used were of analytical grade. The pure compound panchovillin (Figure 1) was isolated from *Erythrina schliebenii* in a previous study [12]. Chitosan (molecular weight 20.2 kDa, degree of *N*-deacetylation 73.5%) was obtained by deacetylation of chitin extracted from the waste shrimp (*Litopenaeus vannamei*) shells collected from Dar es Salaam Fish Market at Kivukoni, Dar es Salaam, Tanzania. Tripolyphosphate (TPP) was purchased from Sigma-Aldrich (Stockholm, Sweden) and used without further purification.

### 3.3. Sample Collection

The shrimps (*Litopenaeus vannamei*) were the source of the collected shell waste materials. The shells (15 kg) were sorted and thoroughly washed with running tap water to remove sand and other impurities. The cleaned shells were dried under the sun for three days affording 290 g of the dried shells. The shells were then ground to afford 286 g of fine powder.

### 3.4. Purification of Chitin

Purification of chitin was carried out in three steps, namely, deproteination, demineralization and decolorization, following the reported procedure with some few modifications [33,34]. In a typical experiment, 100 g of fine powdered shells were demineralized by slowly adding 20% HCl at a ratio of 1 g per 14 mL (*w*/*v*). The mixture was stirred at room temperature until no evolution of gas was observed. The mixture was then filtered and washed with distilled water to a neutral pH. The demineralized powder (60 g) was deproteinated using 1 M NaOH added at the ratio of 1 g per 10 mL (*w*/*v*). The mixture was then heated with continuous stirring at 70 °C for 24 h and then washed with distilled water until neutral to obtain 41 g of crude chitin. The crude chitin obtained was decolorized by soaking in 1% KMnO_4_ for 1 h followed by 1% H_2_C_2_O_4_·2H_2_O at 60 °C for 1 h [35]. The mixture was finally washed with distilled water and dried at 60 °C to obtain 40 g of pure chitin.

### 3.5. Preparation of Chitosan by N-Deacetylation of Chitin

Chitosan (CS) was prepared by using the already established methods, albeit with minor modifications [33,34,36,37]. Typically, 20 g of chitin were soaked in 200 mL of 50% NaOH and heated at 116 °C for 5 h. The mixture was then cooled, and 300 mL of distilled water were added and left to stand overnight. This was followed by filtration, and the residue was washed with distilled water until neutral to obtain 11 g of crude chitosan. The crude chitosan (11 g) was then dissolved in 250 mL of 1% CH_3_COOH (pH 3.03), and the insoluble materials were removed to give a clear supernatant that was neutralized with 2M NaOH, washed to neutral pH and dried at 60 °C giving 9 g of pure chitosan that was transferred to vials and stored in the refrigerator until required for further analysis and application. The solubility of the obtained chitosan was tested in 1% CH_3_COOH by stirring at 1000 rpm under room temperature for 30 min.

### 3.6. Preparation of CS/TPP and PANV-CS/TPP Nanocomposites

Chitosan nanomaterials were synthesized through the ionotropic gelation of chitosan with tripolyphosphate (TPP) [19,38,39]. In an experiment, chitosan was dissolved in acetic acid aqueous solution to give three different concentrations namely, 1 mg/mL (prepared by dissolving 5 mg of chitosan in 5 mL of acetic acid), 2 mg/mL (prepared by dissolving 10 mg of chitosan in 5 mL of acetic acid) and 3 mg/mL (prepared by dissolving 15 mg of chitosan in 5 mL of acetic acid). The concentration of acetic acid in all cases was kept 1.75 times higher than that of chitosan [38]. Furthermore, 2 mL of the 1 mg/mL solution of sodium tripolyphosphate (prepared by dissolving 2 mg of TPP in 2 mL of distilled water) were added dropwise to 5 mL of each of the aforementioned concentrations of chitosan solution under continuous stirring at 1000 rpm for 1 h at room temperature [19,40]. This led to a spontaneous fabrication of chitosan sols. The nanostructured sols were then separated by centrifugation to afford nanostructured chitosan that was freeze-dried, weighed and stored in a refrigerator until required. The PANV-CS/TPP nanocomposites were similarly prepared by ionotropic gelation of CS/TPP with chitosan solutions of 1, 2 and 3 mg/mL being prepared separately [19,38]. The encapsulation of PANV was achieved using 2.5:1, 5.0:1 and 7.5:1 CS/TPP mass ratios to establish the effective CS/TPP ratio that would give better encapsulation efficiency. For further studies, such as the in vitro release kinetics, the in vivo antimycobacterial assay and physical characterizations, PANV-CS/TPP nanocomposites were prepared using the 5.0:1 CS/TPP mass ratio.

### 3.7. Effect of PANV and Chitosan Concentrations on Encapsulation Efficiency

The study on the effect of panchovillin concentration on encapsulation efficiency using different concentrations (1, 2 and 3 mg/mL) of chitosan solution was investigated. Firstly, the blank supernatant chitosan nanomaterials for each chitosan concentration were prepared using 1 mg/mL TPP to serve as the controls during encapsulation. Different concentrations (0.04, 0.08, 0.1 and 0.2 mg/mL) of PANV were separately prepared and added dropwise to each of the chitosan concentrations. The solution was then stirred at 1000 rpm for 30 min to enhance the homogeneity. To prepare PANV-CS/TPP nanocomposites, the crosslinking agent, that is TPP solution (1 mg/mL), was added dropwise to the solution containing chitosan and the test compound. The mixture was continuously stirred at 1000 rpm for 1 h under room temperature. Finally, the supernatant containing the free compound was centrifuged at 3500 rpm for 30 min and filtered using 125 mm filter paper to obtain a clear solution. The effects of the PANV concentrations at different concentrations of chitosan were then studied using a UV-vis spectrophotometer.

### 3.8. Encapsulation of PANV for Bioassay Using Chitosan Solution

The 2 mg/mL chitosan solution was selected for encapsulation of PANV. The PANV-CS/TPP nanocomposites were obtained through the established protocol [19]. The resulting PANV-CS/TPP nanocomposites were centrifuged at 3500 rpm for 30 min under room temperature. The supernatant containing free PANV was collected and filtered using 125 mm filter paper to acquire a clear solution. The clear supernatant solution was analyzed using the UV-vis spectrophotometer to quantify the free compound. The nanocomposites collected were mixed with pure water and re-centrifuged. The supernatant obtained was further analyzed using the UV-vis spectrophotometer for the presence of free natural product. The supernatant was discarded if the natural product was not detected. The nanocomposites obtained were freeze-dried, weighed and stored for characterization, the in vitro release and in vivo antimycobacterial studies.

### 3.9. Encapsulation Efficiency and Loading Capacity

The encapsulation efficiency (EE) of the nanomaterials is the amount of the test compound entrapped in the nanomaterials. It is calculated by taking the ratio of the amount of test compound in the nanomaterials to the amount of the initial total amount of the test compound multiplied by 100% (Equation (1)). The loading capacity (LC) of nanomaterials, on the other hand, is the ability of the nanomaterials to entrap the test compound. The latter is calculated by taking the ratio of the amount of the active compound entrapped to the amount of the nanoformulation multiplied by 100% (Equation (2)) [19,38,41,42]. The process of encapsulation was carried out using 2 mg/mL and 0.1 mg/mL of chitosan and PANV, respectively.

(1) $$\mathrm{EE}=\frac{\mathrm{Total}\;\mathrm{amount}\;\mathrm{of}\;X-\mathrm{Free}\;\mathrm{amount}\;\mathrm{of}\;X}{\mathrm{Total}\;\mathrm{amount}\;\mathrm{of}\;X}\times 100\%$$

(2) $$\mathrm{LC}=\frac{\mathrm{Total}\;\mathrm{amount}\;\mathrm{of}\;X-\mathrm{Free}\;\mathrm{amount}\;\mathrm{of}\;X}{\mathrm{Weight}\;\mathrm{of}\;\mathrm{nanoformulation}}\times 100\%$$

where *X* is the test compound under investigation.

### 3.10. In Vitro Release Studies

The PANV-CS/TPP (22 mg) containing about 0.1008 mg of panchovillin was suspended in a glass bottle containing 100 mL of the receiving medium at pH 7.4. The medium was composed of phosphate-buffered saline (PBS) solution containing 10% ethanol to enhance the solubility of the test compound [43,44,45]. The mixture was then stirred and incubated at 37 °C [19,38]. At appropriate time intervals (1, 2, 4, 6, 10, 22, 34, 48 and 72 h), 5 mL of the sample were withdrawn from the receptor medium and centrifuged at 3500 rpm for 30 min while maintaining the sink condition by replacing the removed sample with fresh medium [19,44,45]. The amount of the test compound released in the supernatant was evaluated using the UV-vis spectrophotometer at a 295 nm wavelength of maximum absorption. The profile for cumulative percentage of in vitro release was plotted using Equation (3) [46].

(3) $$\%\mathrm{Release}=\frac{\mathrm{Released}\;\mathrm{PANV}\;}{\mathrm{Initial}\;\mathrm{PANV}}\times 100$$

### 3.11. In Vivo Antimycobacterial Bioassay Using Galleria Mellonella

The sub-culturing of MIP followed the standard procedure [47]. The *G. mellonella* larvae were inoculated with the sub-cultured MIP by injecting into the hemocoel through the final pro-leg. Bacterial inoculums were prepared from five-day grown cultures in Middlebrook 7H9 broth base containing 0.1% Tween 80, and the turbidity was adjusted to the equivalent of 0.5 McFarland units (approximately 1.2 × 10^8^ CFU/mL). The injection was carried out by opening the last left pro-leg while softly applying pressure to the sides of the larva’s body. The aperture following injection was re-sealed after the removal of the syringe, which led to imperceptible indication of the damage. Incubation was done at the temperature of 37 °C with respect to the controls in order to study the effect of both free PANV and PANV-CS/TPP. The *G. mellonella* larvae were then observed after 24 and 48 h. To investigate further the effect of test bioactive compound on the growth of MIP in the larva’s body, the *G. mellonella* larvae were dissected, and the images were developed to assess the effect of treatment with respect to the controls.

### 3.12. Characterization of Materials

Infra-red (IR) spectra were recorded on the Alpha FTIR spectrometer from Bruker Optic GmgH, equipped with an ATR platinum crystal. The spectrometer was set to perform a total of 25 scans on each sample in the range of 500 to 4000 cm^−1^. The absorption peaks generated during analysis were used for the identification of the structural features of the materials and for the determination of the degree of deacetylation (DD) of the prepared chitosan. The degree of deacetylation was determined by the computation Equation (4) with the amide I probe absorption band and the hydroxyl reference band of 1651 and 3361 cm^−1^, respectively [48].

(4) $$\mathrm{DD}\;=\;\left(100-\frac{{\text{A}}_{1655\;{\mathrm{cm}}^{-1}}/{\text{A}}_{3450\;{\mathrm{cm}}^{-1}}}{1.33}\times 100\right)\;\%$$

Absorption spectra were recorded on a Shimadzu UV/vis-240 spectrophotometer in the range of 200 to 800 nm. The molecular weight of the purified chitosan was determined using MALDI-TOF-MS analysis. The tetrahydrofuran (THF) solution of the chitosan sample and *trans*-2-[3-(4-tert-butylphenyl)-2-methyl-2-propylidene] malononitrile (DCTB) matrix (1:10 *v*/*v*) were mixed in an Eppendorf tube. Then, 0.5 µL of the resulting solution was deposited on two spots on the stainless microtiter format MALDI target and dried at room temperature to produce a solid layer. The MALDI-TOF mass spectrum of chitosan was acquired by 500 laser shots by autoflex when the instrument was operating in the positive ion linear (flight path 1.22 m) mode with an acceleration voltage of 20.04 kV [49].

The XRD analysis was used to characterize the crystalline nature of the chitosan nanostructure. The sample was ground in an agate mortar, resulting in a powder, which was then loaded on the sample holder, compressed and levelled before being subjected for X-ray diffraction analysis. The X-rays generated by a copper anode source were filtered to produce monochromatic radiation that was directed to the sample. Throughout the analysis, the spectra were recorded using CuKα radiation and the 2θ sampling technique. The voltage and current of 40 kV and 40 mA, respectively, were set for the sample analysis, and the scan range was 2° to 70° with a step size of 0.07°. The time, 10s per step, was set, and the sample analysis was completed after three hours. The sample XRD patterns’ data generated were processed using Philips texture software as a pole figure and orientation distribution functions (ODF). The thermal behavior of the prepared materials was determined using the Linseis STP PT-1000 simultaneous thermal analyzer (STA). The samples for analysis were finely ground, and 6 mg of the sample in each analysis were put in the fused alumina, Al_2_O_3_ sample holder and analyzed at the temperature range of 50 to 800 °C at the heating rate of 5 °C/min. The results were plotted with temperature and mass loss. The surface morphology analysis of chitosan, CS/TPP and PAN-CS/TPP materials was performed by using a Zeiss Ultra plus FEG scanning electron microscopy (SEM) and the LEO Ultra 55 SEM.

## 4. Conclusions

The polyphenol panchovillin was encapsulated into the CS/TPP framework by ionotropic gelation. FTIR analysis indicated a successful entrapment of PANV in the nanomaterial. The thermal stability of the nanocomposite was enhanced to 200 °C compared to the pure panchovillin, whose decomposition occurred at 262 °C. The in vitro release profile of panchovillin from the CS/TPP nano-framework was initially fast, up to 42% after six hours, and was followed by a slow release, thereby reaching the maximum release of 81% after 72 h. The in vivo antimycobacterial activity of the pure PANV and the PANV-CS/TPP nanocomposites was comparable at 24 h but different at 48 h. However, it should be noted that the concentration of the bioactive compound in PANV-CS/TPP nanocomposites injected into the larvae was remarkably low (0.289 nM/mg bw), hence proving that the efficacy of PANV was indeed enhanced when encapsulated in the CS nanomaterials. Thus, the results observed in this study are a benchmark for similar studies aiming at improving the efficacy of bioactive natural products.

## Figures and Tables

**Table 1:** In vivo antimycobacterial activity of *Mycobacterium indicus pranii* (MIP)-infected *Galleria mellonella* larvae.

Compound Dose	24 h		48 h	
	Dead (%)	Alive (%)	Dead (%)	Alive (%)
PANV (80 nM/mg bw *)	14	86	29	71
PANV-CS/TPP (0.289 nM/mg bw *)	20	80	60	40
CS/TPP	0	100	0	100

* bw = body weight.
